# Global Assessment of Functioning (GAF): properties and frontier of current knowledge

**DOI:** 10.1186/1744-859X-9-20

**Published:** 2010-05-07

**Authors:** IH Monrad Aas

**Affiliations:** 1 Department of Research, Vestfold Mental Health Care Trust, Tönsberg, Norway

## Abstract

**Background:**

Global Assessment of Functioning (GAF) is well known internationally and widely used for scoring the severity of illness in psychiatry. Problems with GAF show a need for its further development (for example validity and reliability problems). The aim of the present study was to identify gaps in current knowledge about properties of GAF that are of interest for further development. Properties of GAF are defined as characteristic traits or attributes that serve to define GAF (or may have a role to define a future updated GAF).

**Methods:**

A thorough literature search was conducted.

**Results:**

A number of gaps in knowledge about the properties of GAF were identified: for example, the current GAF has a continuous scale, but is a continuous or categorical scale better? Scoring is not performed by setting a mark directly on a visual scale, but could this improve scoring? Would new anchor points, including key words and examples, improve GAF (anchor points for symptoms, functioning, positive mental health, prognosis, improvement of generic properties, exclusion criteria for scoring in 10-point intervals, and anchor points at the endpoints of the scale)? Is a change in the number of anchor points and their distribution over the total scale important? Could better instructions for scoring within 10-point intervals improve scoring? Internationally, both single and dual scales for GAF are used, but what is the advantage of having separate symptom and functioning scales? Symptom (GAF-S) and functioning (GAF-F) scales should score different dimensions and still be correlated, but what is the best combination of definitions for GAF-S and GAF-F? For GAF with more than two scales there is limited empirical testing, but what is gained or lost by using more than two scales?

**Conclusions:**

In the history of GAF, its basic properties have undergone limited changes. Problems with GAF may, in part, be due to lack of a research programme testing the effects of different changes in basic properties. Given the widespread use, research-based development of GAF has not been especially strong. Further research could improve GAF.

## Background

A large number of scoring systems have been developed for psychiatry. The Global Assessment of Functioning (GAF) is known worldwide, has been translated into many languages, and used in many outcome studies [1-3]. In the US, GAF is used for all patients receiving mental health care in the Veterans Health Administration system [4-8]. In Norway, from 2000 onwards, GAF was included in the computerised Minimum Basis Data Set that all mental health services have to report [9,10]. In Denmark, Sweden and in the UK, GAF is also well known [11-13]. The present GAF is found as Axis V of the internationally accepted *Diagnostic and Statistical Manual of Mental Disorders, fourth edition text revision *(DSM-IV-TR). In spite of the fact that it has been recommended for routine clinical use [2], several authors have drawn attention to problems with GAF [3,5,6,9,10,13,14].

GAF covers the range from positive mental health to severe psychopathology, is an overall (global) measure of how patients are doing [15,16], and is intended to be a generic rather than a diagnosis-specific scoring system. GAF reflects a need for more multidimensional information about the patients, rather than diagnosis [14,16], and it measures the degree of mental illness by rating psychological, social and occupational functioning [3,17].

In 1962, the HSRS (Health-Sickness Rating Scale) was published. Studies of the HSRS resulted in a proposal for a new scoring system in the 1970s, the Global Assessment Scale (GAS). Further development led to GAF in 1987. The split version of GAF proposed in 1992 had separate scales for symptoms (GAF-S) and functioning (GAF-F) [3,4,9,10,14,15,17-21]. Internationally, both single-scale and dual-scale systems are in use. In both the single-scale version and the separate GAF-S and GAF-F scales, there are 100 scoring possibilities (1-100). The 100-point scales are divided into intervals, or sections, each with 10 points (for example 31-40 and 51-60). The 10-point intervals have anchor points (verbal instructions) describing symptoms and functioning that are relevant for scoring. The anchor points represent hierarchies of mental illness [3,10,22]. The anchor points for interval 1-10 describe the most severely ill and the anchor points for interval 91-100 describe the healthiest. The scale is provided with examples of what should be scored in each 10-point interval. For example, patients with occasional panic attacks are given a symptom score in the interval 51-60 (moderate symptoms), and patients with conflicts with peers or coworkers and few friends, a functioning score in the interval 51-60 (moderate difficulty in social, occupational or school functioning) [14,23]. The finer grading within intervals provides the possibility of distinguishing between nuances [24], but there are no verbal instructions for this grading found on either of the two scales.

Problems with both the reliability and validity of GAF have been found. Reliability studies show the extreme 20% of raters to account for more than 50% of the spread of scores and deviations can be 20 points or more [3,19]. Overall reliability can be good, but is lower in the routine clinical setting [3,13,15,25-27]. Concurrent validity [1,2,4,8,10,17,25,26,28-34] and predictive validity [8,9,15,17,29,35,36] are more problematic. There are few empirical results for GAF sensitivity [37]. Further development of GAF means work is needed to improve validity and reliability, and to ensure good sensitivity and generic properties.

Properties of GAF are defined in this study as characteristic traits or attributes that serve to define GAF (or may have a role to define a future new GAF). The gaps identified in the present study are defined as properties of GAF where no, or little, research has been performed, with characteristics that suggest further development is likely to have a role for improvement of GAF.

The purpose of the present study was to identify gaps in current knowledge about properties of GAF that are of interest for its further development.

## Methods

### Basic literature search

A literature review [38-40] was carried out. The search was conducted by both hand search and a search of bibliographic databases in several steps (see below). Steps (a) and (b) represent a necessary 'end of the thread' to initiate the literature search.

(a) From previous work, the author had access to literature about relevant issues, namely, literature reviews of scoring systems, which also include information about methodology, other scoring systems, design of questionnaires, and interviews.

(b) Browsing through journals was also performed, which has been recommended as a useful first step before computer search [38]; in the present study, each issue of a set of journals for the period January 2000 to July 2008 was searched *(Acta Psychiatrica Scandinavica*, *American Journal of Psychiatry*, *Archives of General Psychiatry*, *BMC Psychiatry*, *British Journal of Psychiatry*, *British Medical Journal*, *Comprehensive Psychiatry*, *EvidenceBased Mental Health*, *Psychiatric Bulletin*, *Psychiatric Services*, *Social Psychiatry and Psychiatric Epidemiology*, and *The Journal of the Norwegian Medical Association*).

(c) A thorough hand search was performed after identification of publications by steps (a) and (b); their reference lists were hand searched for more literature and by, reading total publications, a search for citations to other studies was also conducted. Each time a relevant publication was identified the same search for new literature was performed. After several rounds of such hand searching, new relevant references became difficult to find and the search proceeded to steps (d) to (g).

(d) A search in PubMed, which used experiences from research on search strategies [39,41-44] was performed. A search was carried out for English language articles from the period January 1990 to July 2008. Search terms were: 'Global Assessment of Functioning OR GAF AND' combined with seven search terms (reliability, validity, sensitivity, literature review, systematic review, psychometrics, methodology) in seven separate searches. A total of 1,599 studies were identified by the PubMed search.

(e) Possible missing publications were controlled for by a search in Google Scholar (for both books and articles) on 25 August 2008, and without limiting the search to a specific time period. The search terms 'Global Assessment of Functioning psychiatry' (used in one common search) identified 162,000 items (mostly publications), and the first 1,000 were screened for relevance. Google Scholar gives information about the number of links to each publication (this is effectively a citation tracking with the most frequently cited publications listed first). The Google Scholar search identified six studies not identified by steps (a) to (d).

(f) A search in The Campbell Collaboration Library of Systematic Reviews on 18 December 2009 was carried out in response to suggestion from the study reviewers. The all-text searches were not limited to a specific time period. Five separate searches were performed (search terms: GAF, Global Assessment of Functioning, psychiatry systematic review, psychiatry literature review, psychiatry review). However, this search identified no relevant studies.

(g) After identification of publications by steps (d) and (e), their reference lists were also hand searched for more literature. New publications that were relevant for inclusion were difficult to find, and the literature search was then considered complete.

### Towards the end of the literature search

The abstracts from steps (d) and (e) were screened with the purpose of identifying literature describing the frontier of knowledge about the properties and modifications/changes of GAF. The frontier of knowledge is the boundary or limit of current knowledge. When this screening started, the researcher was experienced from reading literature from steps (a) to (c). Abstracts were evaluated for inclusion by looking for information on the following issues in relation to GAF: scaling, nature of anchor points, scoring of symptoms and functioning, scoring within 10-point intervals, psychometrics (studies with information on validity and reliability), history of GAF, modifications/changes made, and a more multidimensional GAF. When the screening of abstracts was finished, selected publications were read in their entirety, but it became clear that most of the relevant literature had already been identified by steps (a) to (c).

The final set of selected publications is the reference list of the present study. Included publications are original research papers, books, articles, letters to the editor and book reviews.

### From the frontier of current knowledge to gaps in knowledge

The contribution of each selected publication to the frontier of current knowledge was summarised [38], and analysis was then performed to identify gaps in knowledge that were considered to be of interest for further development of GAF.

## Results

The literature review identified four main categories (each with a number of subcategories) of properties of GAF that were important in relation to its further development: (1) scaling; (2) the anchor points of GAF; (3) scoring within 10-point intervals; and (4) the number of scales.

The presentation of properties in the present study does not require any distinction between the single-scale and dual-scale GAF. When the single scale is used, 'whichever is the worse' of the symptom and functioning values is the single value recorded (according to the manual for DSM-IV-TR).

### Scaling

Problems concerning measurement and scaling are fundamental in science and decisive for evaluation of interventions in health care. Scaling means quantifying qualities by assigning numbers [45]. For psychiatry, scaling has been, and will continue to be, central to its development [22,46-49]. The choice of rating scale is not indifferent: problems in scaling can be due to properties of the rating scale [50,51].

### Continuous or categorical scale

A continuous scale has no steps and does not force the respondent to answer in specific categories [52]. In GAF, a continuous scale (finely graded with 100 points) has been preferred to a discrete scale. With good reliability, sensitivity using continuous scales can be good for detecting change and differences. Statistical testing can show statistically significant differences for samples with small differences in the severity of illness. Continuous scales may also be applied to defining threshold values for assigning diagnoses. It is plausible that symptoms and functioning are more continuous in nature than mental illness itself. Error of measurement for such a finely graded scale may also mask a possible discontinuity of mental disorders. In GAF, the anchor points are ranked, but it is open to question whether the anchor points (with key words and examples) really constitute a natural continuum.

An alternative to a continuous scale is classification into categories with verbally formulated inclusion criteria for each category. The internationally well known symptom checklists are clear examples [53]. The simplest way of scoring symptom and functioning items is to score present or absent [24], but scorers can be capable of making more accurate judgements, for example by using a Likert-type scale with five categories, ranging from not present to present to a marked degree [46,54]. The items of a symptom checklist must be relevant for the disorder(s) to be studied (that is, a generic scale requires an all-inclusive set of symptoms). If mental disorders can be said to develop in stages, disease-staging systems could be chosen [55-57]. The categories are then the stages of the disease-staging system. GAF is not without similarity to categorical scales (that is, the 10 anchor points can be viewed as categories). However, it is not really known whether mental disorders are continuous or discrete in nature [49,58-60].

Gap in knowledge: the development of GAF has little basis in general research on what is best for a global functioning scale (that is, a continuous or categorical scale). Little research has been performed directly on GAF concerning whether a continuous or categorical scale is better.

### Visual scale

A VAS (visual analogue scale) is a line with anchor points at each end to indicate the extremes. The scorer marks a point on the scale indicating the severity of the phenomenon. The scored value is the distance from the point to the scale's lower end. The VAS has been used successfully in psychiatry, but there is no conclusive evidence that it is better than categorical scales and it takes more work to analyse [46,51,53,54,61,62]. When a VAS is equipped with descriptive anchor points along the line, it becomes more similar to a scale that could work as a visual scale for GAF. Technologically, it is possible to computerise scoring on a VAS by setting a mark on the screen's digital line, so the computer calculates the distance from the lower end of the line.

Gap in knowledge: we do not know whether scoring directly on a visual scale improves scoring for GAF and whether computerisation of such scoring gives better results (for example, improved reliability). If a visual scale is equipped with descriptive anchor points along the line, we do not know which anchor points will be best, how many anchor points should be used, and where along the line the anchor points should be located.

### Scales and further treatment of data

Raw data from scaling and measurement often undergo statistical analysis. For such analysis, it is relevant to distinguish between four types of scales: nominal, ordinal, interval and ratio scales. Both nominal and ordinal scales are well known in psychiatry and GAF is an example of an ordinal scale. This has consequences for further treatment of data. We cannot say, for example, that a 5-point change in GAF from 38 to 43 means the same change in severity as that from 68 to 73. Mean GAF at the start of treatment minus mean GAF at the finish, for sample A, cannot be said to be larger than the same change for sample B, in spite of sample A clearly having a larger numerical difference than sample B [22]. Similarly, it is not entirely correct to add individual scores and divide by the number of individual scores to obtain the mean value. For psychiatry, it is difficult to develop a mental health scale that reaches the level of a real interval or ratio scale, but it is quite common to see GAF data treated as something more than ordinal data. In some research projects, collected raw data for GAF are merged into a limited number of categories [15,63]. A simple version of this is to dichotomise the level of functioning into 'superior to fair' and 'poor to grossly impaired' [64]. Some authors have merged their raw data into more categories (from three to seven [15,63,65-67]). It would be expected that such categorisation of a raw data set is important for conclusions drawn when the data are treated statistically. For a single scale GAF 'whichever is the worse' of an individual's symptom and functioning values is the GAF score [68]. Also, when scoring is performed on two separate scales (GAF-S and GAF-F scales), sometimes only one score is recorded. In principle, this could be the lower, average or higher of the two scores. As GAF-S and GAF-F score different dimensions, giving just one figure is open to criticism and also means loss of information.

Gap in knowledge: when GAF data are treated as something more than ordinal data it is possible that the resulting error is small, but there has been little testing of whether the error is of any practical interest. Similarly, the error resulting from merging raw data into broader categories, and the use of just one score in GAF, have not been subjected to much scrutiny.

### The anchor points of GAF

The use of symptoms and functioning as an expression of severity of illness is well known. Furthermore, psychiatric diagnoses express differences in severity, and severity can also include factors such as stage of development of the illness, intensity (for example, frequency and duration of periods with symptoms over a time period), and comorbidity [69-72].

### The nature of anchor points

The 10 anchor points (with key words and examples of symptoms and functioning items) give a general idea on what to stress in scoring GAF. The use of examples is important and is likely to improve assessment [73]. In Hall's 'modified GAF' a greater number of criteria for scoring are found [28]. Items used in different symptom and functioning scoring systems are different; in further work with GAF, ideas for the best subset of items can be drawn from the literature on symptom and functioning scoring [2,22,53,74,75].

The anchor points should give descriptions that are sufficiently close to what the clinician observes. Validity may be improved with concrete anchor points [8]; the anchor points of GAF could be worked out with more examples. As the anchor points are ranked, we are dealing with symptoms (and also functioning) as being something unidimensional, but ranking of items is especially difficult when they are each very different.

Gap in knowledge: in the history of GAF, little change is found in the character of anchor points, key words and examples. We do not know if other anchor points, with other key words and examples, would give a better GAF. We do not know if other expressions of severity (such as stage of development of the illness, intensity, and comorbidity) could be included as scoring criteria. There has been little analysis of whether all the rankings of anchor points are correct. We have little information about potential differences in the validity and reliability for low and high scores.

### Symptoms

The current symptom anchor points were generally assigned in earlier stages of development that led to the present GAF, but much symptom research has been performed since then. Symptom checklists can include questions about behavioural and somatic symptoms, and positive and negative feelings of well-being [22,76]. Asking about both positive feelings of well-being and somatic symptoms makes the checklist more objective; sensitivity and specificity can be good, and the intent of the measurement is concealed [22]. As patients can have more than one symptom, with different types and degrees of development, assessments of illness severity based on such symptom clusters seems logical. Many symptoms in psychiatry have two aspects: form (for example, auditory hallucination) and content (for example, the person is told to do something) [77]. In symptom-scoring systems, symptom content has been largely ignored, but perhaps it should not be [73].

Gap in knowledge: the considerable body of symptom research has played a limited role in the development of GAF. It is possible that anchor points, key words and examples for anchor points could be improved by learning from symptom research. Symptom clusters, with different degrees of severity for each symptom, have been little evaluated for scoring in GAF. A change in symptom anchor points could have an effect on scoring within 10-point intervals. There has been little evaluation of symptom content as a criterion for scoring illness severity.

### Functioning

A large number of indices of functioning have been constructed [17,22,74,78]. Functional status can be defined as the degree to which an individual is able to perform socially allocated roles free of mentally (or physically) related limitations [74]. A measure of functioning requires decisions about: which type of functioning should be scored (for appraisal of overall functioning, several types of functioning should be scored, for example difficulties with participation in working life, daily activities, and social relationships); how to grade each type of functioning; and whether an aggregate measure can be made (that is, the total score expressed with one figure).

When functioning is scored in psychiatry, impairments with a somatic background should be excluded [23,26], but GAF-F values can be the result of combined mental disorder and somatic disease; some illnesses have a psychosomatic background and somatic diseases can be followed by a psychological reaction. When scoring is carried out for longer time periods, such as 1 year, it can be difficult to attribute functioning values to mental status alone [17].

When a GAF-F value has been assigned, this should mean that the patient is not able to perform tasks that are higher on the scale, but early support can be associated with improved functioning measured by GAF [30] (that is, support from healthcare, or family and friends). A patient having problems with functioning at work can achieve a better score by moving to a new job. An advantage with scoring of functioning is that it can be more easily applied across diagnostic groups [35].

Gap in knowledge: the considerable international research on functioning has played a limited role in the development of GAF. It is possible that anchor points, keywords and examples for anchor points, and scoring within 10-point intervals could be improved by learning from research on functioning. Little analysis has been carried out of different combinations of types, number, and grading of functioning anchor points, and further work is needed to determine the optimal reliability, validity, sensitivity and generic properties of the anchor points.

### Positive mental health

In psychiatry, there is a preoccupation with mental illness, but less interest in positive mental health [70,79]. Positive and negative feelings are not simply opposite ends of a single-dimension scale [22]. It could be discussed whether the scoring of GAF should include factors such as life satisfaction, positive quality of life, psychological well-being, and even physical fitness [70,71,74]. Inclusion of questions about 'positive mental health' may be important for prediction of the ability to improve after an episode of mental illness.

Gap in knowledge: a further development of GAF could include a search for indicators of positive mental health. It is possible that inclusion of positive health factors will improve the choice of 10-point interval, and the scoring within 10-point intervals. Different combinations of the types, number and grading of positive health factors have not been analysed to obtain the best possible reliability, validity, sensitivity and generic properties. In addition, there has been little assessment of different combinations of positive and negative feelings in the scoring.

### Prognosis

The present GAF has limited value for assessing prognosis [63], and other systems predict prognosis better [25,36,53]. Prognosis is definable as a part of the severity of illness. A patient who is severely ill with a good prognosis can then be scored more highly than a patient who is less severely ill with a poor prognosis. Prognosis can be related to the patient's resources and not just the patient's problems and is more dependent on diagnosis and symptoms than impairment ratings: the highest level of functioning for a time period is more important for prognosis than the lowest, and substance abuse plays a role [15,70,71,74].

Gap in knowledge: prognosis has not been much considered as a criterion for scoring in GAF. In the further development of GAF, prognosis may be considered as a criterion for scoring.

### Generic properties

In the DSM-IV-TR, there is an overlap between criteria for diagnoses and criteria for GAF scoring. A relationship with diagnoses can be expected for GAF [15,26,32,34,63,80,81], but DSM is a multiaxial system [32] where each axis is intended to add information. In their work with GAS, Endicott *et al*. [18] wanted to remove all diagnostic criteria. A different strategy would be to develop different criterion sets for different diagnoses (for example, for dementia and depression). The use of diagnosis-specific symptoms and functioning criteria for GAF scoring could improve the generic properties of GAF.

GAF was intended to be used for both for adults and children [14], but a specific version for children has been developed. The Children's Global Assessment Scale has anchor points that are especially relevant for children [82].

Gap in knowledge: reviews showing strengths and limitations of GAF's generic properties are difficult to find. Such reviews could form the basis for change in anchor points, for example by adding criteria that are relevant for diagnoses where scoring of GAF is difficult due to lack, or low relevance, of criteria. Reviews of GAF's generic properties could also give information that is important for construction of specialised GAF scales for patient groups that are poorly covered by the present GAF.

### Exclusion criteria

The anchor points are generally inclusion criteria for scoring in 10-point intervals. Little work has been performed to identify exclusion criteria for scoring in each interval. An example would be identification of symptoms (or grading of symptoms) that exclude scoring in the GAF-S interval 51-60 and make the interval 41-50 preferable. Proposing that the anchor points of neighbouring 10-point intervals are exclusion criteria may be too simple an answer.

Gap in knowledge: in the history of GAF, little work has been performed to elucidate exclusion criteria for scoring in each interval. A further development of GAF could include a search for specific exclusion criteria.

### Extremes of the GAF

The GAF scale identifies the lowest and highest levels for a hierarchy of mental illness. The choice of anchor points at the endpoints is decisive for the variation in possibilities of a phenomenon, as endpoints can influence which score is given [62]. In scoring of morbidity, perfect health often marks one extreme. In GAF-S, the other extreme is persistent danger of severely hurting themselves or others, and in GAF-F it is persistent inability to maintain minimal personal hygiene. In a disease-staging system, death was chosen as the lower endpoint for a number of psychiatric conditions [55]. However, not all health states can be placed upon a continuum bounded by the anchor points 'perfect health' and 'death' [62]. Patients themselves can consider some conditions worse than death [52,62]. In the Kennedy Axis V's subscale for psychological impairment, criteria have been added to the GAF criteria, such as 'totally insensitive to the feelings and need of others' (the lowest interval) [83]. The first step in work with a scaling instrument should be to define its endpoints.

Gap in knowledge: we know little about the influence on GAF scores of using other anchor points at the endpoints of the scale.

### Number of anchor points

The 100 scoring possibilities in GAF and the low detail of verbal instructions are in conflict with each other. Equipping GAF with a higher number of anchor points could be considered [10]. In general, the middle range is frequently used in psychiatry, and more elaborate verbal instructions for the middle range could be considered [82]. For newly admitted inpatients, higher scorings are rarely used, which gives relevance to having more anchor point for the lower range [18]. In community studies, the upper part of the scale is most relevant, and so the question of having more anchor points for the upper range also comes up. When scoring of GAF is computerised, links can be visible on the screen and clicking on these links gives more detailed information (for example, for scoring newly admitted inpatients and for community studies).

Gap in knowledge: systematic testing of different changes in the number of anchor points (and their distribution over the total scale) to obtain a better GAF is difficult to find in the history of GAF.

### Scoring within 10-point intervals

Endicott *et al*. [18] and the manual for DSM-IV-TR give instructions for scoring within 10-point intervals, but instructions are limited. In practice, clinicians tend to score around the decile, or mid-decile, divisions of the scale [16]. When information for a more accurate score is lacking, intermediate scores in the deciles are chosen [21,51].

For improved scoring within the 10-point intervals of current GAF, three tools can be considered: more detailed verbal instructions, development of categorical scales for scoring within the 10-point intervals, and the number of criteria met to decide a score within a 10-point interval.

### More detailed verbal instructions

More detailed verbal instructions could be developed with the intention of improving scoring within 10-point intervals, that is, more anchor points (more keywords and examples) specified to improve scoring within 10-point intervals.

### Development of categorical scales

Categorical scales could be developed to improve scoring within 10-point intervals. This means grading of anchor points (with key words and examples of symptoms and functioning items). Categorical scales often have five categories, such as 'very marked', 'marked', 'neither marked nor weak', 'weak' and 'very weak'. Although functioning scored by a 5-point scale can have good reliability [84], the optimum number of categories may be five to seven, or more [24,46,50,51,54].

### Number of criteria met

An alterative procedure for scoring within 10-point intervals is found in the 'modified GAF' [28]. The number of criteria met is used, for example for the interval 41-50: when one criterion is met the score should be 48-50 and when two criteria are met the score should be 44-47.

Gap in knowledge: in the history of GAF, systematic work to improve scoring within 10-point intervals is limited. This also applies to evaluation of categorical scales for the purpose. Such application of categorical scaling would require consideration of the nature and number of categories.

### The number of scales

When GAF is scored according to the instructions in the DSM-IV-TR, only one figure is given, but both symptoms and functioning are assessed. However, the recording of only one figure means there is a lack of knowledge about which dimension is represented. Patients can present a complexity that is better described by having two scales (separate GAF-S and GAF-F scales) [10,17,26,35,85].

### GAF with two scales

Reliability and validity studies for both GAF-S and GAF-F scales exist, but there are relatively few [2,8-10,15,26,30]. In psychiatry, symptoms and functioning are often closely related [15,17,26,63], but have been proposed to deviate frequently enough to recommend measuring both in outcome studies [17,35]. Functioning can improve without a corresponding symptom improvement and vice versa [35]. GAF-S and GAF-F can be correlated with r = 0.61 [10]. When GAF-S scores share more variation with other measures of symptoms and GAF-F scores share more variation with other measures of functioning [10], this suggests that GAF-S and GAF-F represent different aspects of a patient's condition. Few studies have focused on concurrent validity of GAF-S and GAF-F separately, but the association between GAF-F and other types of functioning may be low [10,15,30,63]. In general, we have little empirical knowledge about the advantage of separate scores for symptoms and functioning, for example, for assessment of treatment need and measurement of outcome [10]. The clinical significance, when GAF-S and GAF-F are clearly different, has also been little explored.

Gap in knowledge: we know little about the advantage of using GAF with symptom and functioning scales separately. The symptom and functioning scales of GAF should score different dimensions, but the scores should still be correlated. Search for the right combination of definitions of GAF-S and GAF-F is limited. More study should be performed of reliability and validity for both GAF-S and GAF-F scales individually.

### GAF with more than two scales

In the latest version of the DSM (DSM-IV-TR), two extra scales were provided for further study: the Global Assessment of Relational Functioning Scale (GARF) and the Social and Occupational Functioning Assessment Scale (SOFAS). The Mental Illness Research, Education & Clinical Center (MIRECC) GAF has three scales: for symptom severity, occupational functioning, and social functioning [8]. In the Kennedy Axis V, the seven subscales provide a broad profile of the patient [83]. GARF, SOFAS [5,26,29,86], MIRECC GAF [8], and Kennedy Axis V [83] all make more information available to the clinician. If the number of scales is increased, there may be a longer learning time for the scoring method, scoring becomes more time consuming and less easy to use, with analysis of the results becoming more complex (for example for outcome). International diffusion of these scales has been modest.

Gap in knowledge: the advantage of a GAF split into two scales should be investigated more thoroughly before discussing a system with more than two scales. Research on GAF with more than two scales is limited. For example, more study of reliability and validity is necessary, as well as studies of what can be gained and lost by using more than two scales. It seems premature to let such systems replace the current GAF.

### Further development of GAF

For work with a new GAF, some overall goals can be formulated: (1) the scale should continue to cover the range from positive mental health to severe psychopathology; (2) it should continue to be a global measure for how patients are doing; (3) the generic properties should be improved; (4) a new GAF should add information compared to the other axes of the DSM-IV-TR; (5) reliability should be improved or at least not reduced; (6) validity should be improved; (7) sensitivity should be analysed, compared to other scaling methods, and found to be good enough for the purpose; (8) the new system should make sense to clinicians; and (9) scoring should be fast and easy. The goals are ambitious, but not necessarily impossible to combine.

Methodology studies of the design of questionnaires demonstrate the significance of variation in instrument properties for scoring results [50]. The design of scoring instruments for psychiatry shows the same importance of instrument properties for the scoring result [22,24,58,74]. In the historic development of GAF, little study of systematic variation in system properties has been carried out. The study by Hall [28] could have been a start (showed that change in properties can improve GAF), but it has been little followed up. The significance of the gaps in knowledge is an empirical question that can be investigated. Many alternative forms of a new GAF could be examined (with both with major and minor changes). It is difficult to forecast which changes are likely to provide the most significant improvements. Researchers should be aware that even seemingly minor changes can have a major impact [87]. Reliability and validity are connected [10]. For example if validity is improved by a change in the properties of an instrument, reliability may change (with uncertain direction).

The many application possibilities of GAF have not been widely studied. For GAF to function well in different applications, different changes may be required. Psychometric characteristics are not properties of an instrument *per se*, but rather properties of an instrument when used for a specific purpose with a specific sample [88].

For a new GAF, scoring should be completely computerised. The electronic patient record makes new quality assurance methods possible. For example, some diagnoses are incompatible with high GAF scores. If such a diagnosis has been given, a warning could pop up on the screen if too high a GAF score is given. A correlation is expected between what is scored in a symptom checklist and GAF scoring. A warning could pop up on the screen if this correspondence is lacking.

Construction of health scales requires much work. A new GAF should be subjected to rigorous testing of validity and reliability. Work with a scoring instrument is not complete until it has been tested in a pilot study [52].

## Discussion

### Methodology

The starting point of the present study can be defined as a systematic review [41,43]. The study satisfies several important criteria for review articles, such as defining the problem, informing the reader of the status of current research, identifying gaps and suggesting the next step [89].

An encompassing hand search of literature was conducted because it was considered that some relevant publications were likely to be found in publications that are not included in PubMed (for example, methodology literature about scaling in general, and about questionnaires and interviews), but there is a suggestion that studies that are difficult to locate tend to be of lower quality [41]. A combination of searching reference lists and reading publications has been considered the most thorough way of hand searching [90]. The search in PubMed and Google Scholar revealed that most of the publications were already identified by the thorough hand search (step (c) in Methods) and so the present study confirms the opinion that hand search still has a role to play [90,91]. It is not a matter of course that PsycINFO gives better search results than PubMed, but the opposite may result [92-94]. PubMed includes more than 500 psychology-related journals [95]. The search in The Campbell Collaboration Library of Systematic Reviews added no new studies, but methodology studies show that systematic reviews can be identified with high reliability in PubMed [39,42,43]. The citation tracking in Google Scholar is not completely reliable (when it comes to listing the most frequently cited first), but the screening of the first 1,000 results represents a thorough Google Scholar search. The searches in PubMed and Google Scholar are reproducible. Few new perspectives were added by the literature search from steps (d) and (e). A stage was reached where new perspectives could not be identified by reading more publications; this situation is described by the term 'saturation' from qualitative research. It is not considered likely that publications that could have changed the results were missed as a result of the search process. The design and conduct of the present study protected against bias [40,41].

### Why improve GAF?

The history of GAF does not show the research-based development of GAF to be especially strong, particularly in the context of its widespread use. In light of the weaknesses discussed, it might be tempting to conclude that GAF should not be used, but existing scales can be dismissed too lightly [51]. A generic and global scoring system, such as GAF, that covers the range from positive mental health to severe psychopathology has advantages for clinical practice (for example, routine quality assessment of treatment, supplementing scales that give more detail) [54], research (for example, comparison of treatment outcome across diagnoses), and policy and management levels (for example, allocation of resources, measurement of case mix in psychiatric organisations).

### GAF properties and gaps in knowledge

Researching the frontier of current knowledge and gaps in knowledge is a well known starting point for any study. Existing international research on GAF is characterised by researchers paying attention to some aspects (for example reliability), but there is less evidence of well thought out overall research programmes where different properties are systematically changed and tested in order to obtain an optimal system. In such research, independent variables can be different changes in properties, and dependent variables measures of reliability, validity and sensitivity. As GAF is intended to be a generic system, the work could be performed for different diagnostic groups. Although Hall [28] showed that changes in properties can improve GAF, it is not a matter of course that research where properties are changed results in an improved system. The simplicity of GAF is an advantage and a future GAF could become more complex. The potential gains with an improved GAF should be balanced against the consequence of a more time-consuming scoring for each patient (that is, a reduction in total capacity for the mental health service). Comparison between a new GAF and the current GAF will not necessarily show scores that are directly comparable [96]. This may be a problem for comparison of results from different studies, meta-analyses and use of historical data.

Of the many properties of GAF, some are especially relevant for reliability and sensitivity (continuous or categorical scale, scoring performed directly on a visual scale, the number of anchor points, and scoring within 10-point intervals). If reliability is too low for assessment of change for the individual patient, this does not mean that scoring is useless because GAF can be used to measure changes at group level [13]. The character of anchor points is fundamental for validity. To construct a scale, knowledge of the phenomenon to be studied is necessary. The determinants for symptoms and functioning are highly complex. The question can be asked, has research sufficiently defined the nature of psychiatric illness to obtain a severity of illness system that functions well?

### Factors other than properties

The present study has focused on properties of GAF, but other factors can also play a part in choice of GAF value. Factors that have not been treated here include: (1) characteristics of the process of scoring, for example characteristics of the patient interview (such as time on patient interview, structured interviews with which questions, formulated and ordered in which way), time period to consider for scoring (present status, last 3 months, and so on), and who should score (for example, individuals, groups, independent scorers); and (2) characteristics of the interviewer, cultural factors, training and motivation [9,10,13-15,17,34,46,49,50,54,82,86].

## Conclusions

The history of GAF reveals much evidence of continued use of the properties that were developed early and little evidence of further development of the instrument itself. The present study has identified a number of gaps in our knowledge about GAF. Further work should focus on these gaps and requires a research programme that is based on an overview of what is needed for further development. For a new GAF the advantage of computerisation of scoring should be exploited.

## Competing interests

The author declares that they have no competing interests.
